# Photosynthesis in the fleeting shadows: an overlooked opportunity for increasing crop productivity?

**DOI:** 10.1111/tpj.14663

**Published:** 2020-02-24

**Authors:** Yu Wang, Steven J. Burgess, Elsa M. de Becker, Stephen P. Long

**Affiliations:** ^1^ Carl R Woese Institute for Genomic Biology University of Illinois at Urbana‐Champaign Urbana IL 61801 USA; ^2^ Department of Plant Biology University of Illinois at Urbana‐Champaign Urbana IL 61801 USA; ^3^ Department of Crop Sciences University of Illinois at Urbana‐Champaign Urbana IL 61801 USA; ^4^ Lancaster Environment Centre Lancaster University Lancaster LA1 4YQ UK

**Keywords:** photosynthetic induction, non‐photochemical quenching, NPQ, food security, soybean, wheat, photosystem II, photoinhibition, stomata, crop breeding, leaf canopy, Rubisco activase

## Abstract

Photosynthesis measurements are traditionally taken under steady‐state conditions; however, leaves in crop fields experience frequent fluctuations in light and take time to respond. This slow response reduces the efficiency of carbon assimilation. Transitions from low to high light require photosynthetic induction, including the activation of Rubisco and the opening of stomata, whereas transitions from high to low light require the relaxation of dissipative energy processes, collectively known as non‐photochemical quenching (NPQ). Previous attempts to assess the impact of these delays on net carbon assimilation have used simplified models of crop canopies, limiting the accuracy of predictions. Here, we use ray tracing to predict the spatial and temporal dynamics of lighting for a rendered mature *Glycine max* (soybean) canopy to review the relative importance of these delays on net cumulative assimilation over the course of both a sunny and a cloudy summer day. Combined limitations result in a 13% reduction in crop carbon assimilation on both sunny and cloudy days, with induction being more important on cloudy than on sunny days. Genetic variation in NPQ relaxation rates and photosynthetic induction in parental lines of a soybean nested association mapping (NAM) population was assessed. Short‐term NPQ relaxation (<30 min) showed little variation across the NAM lines, but substantial variation was found in the speeds of photosynthetic induction, attributable to Rubisco activation. Over the course of a sunny and an intermittently cloudy day these would translate to substantial differences in total crop carbon assimilation. These findings suggest an unexplored potential for breeding improved photosynthetic potential in our major crops.

## Significance Statement

Improved photosynthetic efficiency is a means to achieve the sustainable crop yield increases that are a projected need for ensuring global food security over the next 30 years. Improving photosynthetic efficiency during fluctuating light in the field is shown from theory and from measured variability within crop germplasm a potential route to breeding higher efficiency.

## Introduction

The improvement of crop photosynthesis for yield increases has focused on rates of leaf CO_2_ uptake at light saturation (*A*_sat_) and under constant light. No correlation between variation in *A*_sat_ and yield has been found across a selection of modern *Triticum aestivum* (wheat) accessions (Rawson *et al.*, 1983). Furthermore, wild ancestors of wheat showed higher *A*_sat_ values than elite cultivars (Dunstone *et al.*, 1973). Such influential early findings led to skepticism that photosynthesis can be improved in crops, and this view persists for some today, despite evidence that bioengineered increases in steady‐state photosynthesis do correspond to significant increases in productivity (Köhler *et al.*, 2016; Sinclair *et al*., 2019; South *et al.*, 2019). Under field conditions, however, the light in a crop canopy is rarely constant. Fluctuations result from intermittent cloud cover but more importantly from the continuous change in the angle of the sun over the course of the day, causing intermittent shadowing within the canopy by overlying leaves and other plant structures (Zhu *et al.*, 2004; Wang *et al.*, 2017b). Might we have overlooked a major opportunity by focusing on steady‐state photosynthesis?

A dynamic light environment affects photosynthesis in two main ways. First, a leaf in the shade of another receives about 1/10th of the light of a leaf in full sun (Zhu *et al.*, 2004). These periods of full sun and of shade may last from seconds to hours (Zhu *et al.*, 2004; Deans *et al.*, 2019; Tanaka *et al.*, 2019). Using reverse ray tracing, and assuming a random distribution of leaves, Zhu *et al. *(2004) showed that even on a clear day, leaves in a static canopy will experience over 20 sun–shade–sun transitions. When leaves move into full light after a period of shade, the leaf CO_2_ uptake rate (*A*) does not instantly reach its maximum value, but rises gradually over several minutes to approach a new steady state. This has been termed photosynthetic induction, and it causes a substantial reduction in the efficiency of carbon fixation (Yamori *et al.*, 2012; Soleh *et al.*, 2017; Taylor and Long, 2017; Salter *et al.*, 2019). Second, leaves in full sunlight receive more energy than they can use, leading to the activation of non‐photochemical quenching (NPQ), which dissipates excess energy as heat. This reduces the production of oxidizing radicals via excited‐state chlorophyll molecules that would damage the photosynthetic apparatus. Similarly, in a sun–shade transition there is a loss of potential CO_2_ assimilation. This is because various components of NPQ have relaxation half‐lives ranging from seconds to hours, and therefore the leaf continues dissipating light as heat even after it is moved into the shade, where it could use all of the light received in photosynthesis. This forces *A* below the steady‐state level that it will eventually resume. Practical proof of the importance of this was shown with a bioengineered increase in the rate of dissipation of NPQ during sun–shade transitions. This resulted in a doubling of *A* in fluctuating light and a significant increase in *Nicotiana tabacum* (tobacco) productivity in replicated‐plot field trials (Kromdijk *et al.*, 2016). Many NPQ components have been identified so far, such as: energy‐dependent quenching, qE (Krause *et al.*, 1982); zeaxanthin‐dependent quenching, qZ (Dall'Osto *et al.*; 2005; Nilkens *et al.*, 2010); chloroplast relocation‐dependent quenching, qR (Cazzaniga *et al.*, 2013); state transition‐dependent quenching, qT (Nilkens *et al.*, 2010); photoinhibition quenching qI; and photoinhibition‐independent sustained quenching qH (Malnoe *et al.*, 2018). However, the relative contribution of these processes may depend on the conditions and species.

It has been argued that evolution and breeder selection would have already optimized photosynthesis in our crops (Winer, 2019). Two factors, with respect to photosynthesis in fluctuating light, counter this argument. First, the ancestors of our major crops evolved in relatively open habitats that could support the production of few leaves, hence both shading and self‐shading would have been rare. For example, in the case of *Glycine max* (soybean), the wild ancestor is an annual twining vine that climbs on other stems and spaces its leaves to largely avoid shading. In contrast, modern elite cultivars have been bred as bush forms, grown at densities where they will form 5–7 m^2^ of leaf area over every 1 m^2^ of field. In essence, the leaves of what were sun plants by origin are now grown so that most leaves are shaded or intermittently shaded. Given the speed at which planting density has increased for our major crops, there is good reason to expect that neither evolution nor breeder selection have kept pace. Indeed, two major crops have now been shown to fail to adapt to shading to optimize canopy photosynthesis (Pignon *et al.*, 2017). Second, current [CO_2_] and light levels are co‐limiting to photosynthesis in C3 crops. Atmospheric [CO_2_] has risen from the 220 ppm average of the past 25 million years to 407 ppm in 2018, with half of that increase occurring in just the last 60 years. This means that light has become progressively more limiting and CO_2_ has become progressively less limiting, strongly affecting photosynthetic efficiency in the shade (Long *et al.*, 2004). Again, it is unlikely that there has been sufficient time for any adaptation to this change. This may be reflected in the large variation in speeds of induction on shade–sun transitions within the germplasm of *Manihot esculenta* (cassava), *Oryza sativa* (rice), soybean and wheat (Soleh *et al.*, 2017; De Souza *et al.*, 2019; Salter *et al.*, 2019; Acevedo‐Siaca *et al.*, 2020). For example, the between‐accession variation in CO_2_ assimilated during the induction was three times that of the steady‐state assimilation in cassava (De Souza *et al.*, 2019), suggesting that optimization of photosynthesis in fluctuating light has not occurred.

As a result of fluctuations in light the leaf transiently forgoes potential assimilation compared with what could be achieved with an instantaneous response of photosynthesis (Zhu *et al.*, 2004; Taylor and Long, 2017; Deans *et al.*, 2019). Over the course of a day, how much potential photosynthesis does a crop canopy forgo? Two recent estimates predicted a daily loss for wheat of up to 15–21% (Taylor and Long, 2017; Salter *et al.*, 2019); however, these estimates did not use realistic canopy models, simulated only small parts of the canopy and did not consider the combined effect of shade–sun and sun–shade transitions. More accurate assessments of these efficiency losses during light fluctuations require the representation of actual crops and the spatial and temporal dynamics of lighting across all leaves in the canopy.

Using soybean as an example, here the structure of an actual canopy of an elite cultivar is combined with forward ray tracing to predict the spatial dynamics of lighting across the entire canopy throughout the course of both a clear sky and an intermittently cloudy day. This dynamic lighting is combined with kinetics of both induction of photosynthesis and NPQ relaxation to quantitatively review the losses that result compared with an instantaneous return to steady‐state photosynthetic rates on light transitions. To simplify the simulation, we divided the components of NPQ dynamics into two groups: short term, ≤30 min (STNPQ), and long term, >30 min (LTNPQ). The variation measured in both induction and STNPQ relaxation across parental lines of a nested association mapping (NAM) population of soybean was used to assess their value for breeding for increased speeds of adjustment to light fluctuations.

## Results

**Figure 1:**
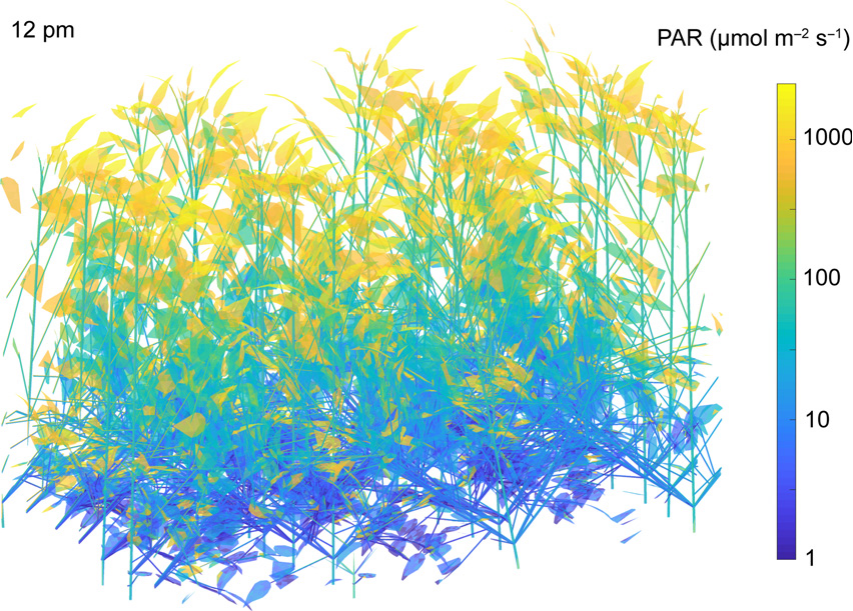
Modelled *Glycine max* (soybean) canopy structure and lighting (PAR= Photosynthetically active photon flux), predicted from ray tracing for a clear sky on 20 August, at Champaign, Illinois, USA (40.11°N). Colors indicate the spatial heterogeneity of the intensity of the absorbed light at noon.

**Figure 2:**
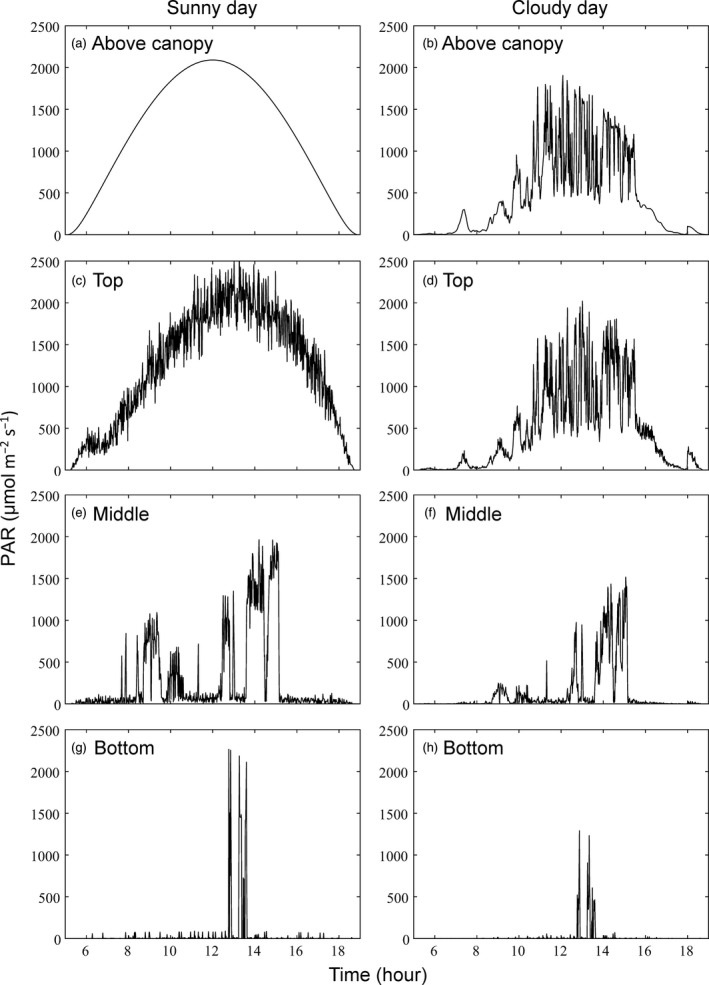
Panel (b) shows the recorded incident photosynthetically active photon flux on 20 August 2019, above the soybean canopy of Figure 1, which was an intermittently cloudy day. Panel (a) shows the predicted light intensity for the same day based on sun–earth geometry and an atmospheric transmittance of 0.85, assuming a cloud‐free clear‐sky day. The diurnal light absorption simulated for single pixels on the leaves in the top, middle and bottom of the soybean canopy on the sunny day are shown in panels (c), (e) and (g), respectively, and for the cloudy day in panels (d), (f) and (h), respectively.

The light absorption of a soybean canopy was simulated for a clear sky (sunny) and intermittently cloudy (cloudy) day in August using the ray‐tracing algorithm (Figure 1). The diurnal light absorption for all points in the canopy was simulated over the daylight hours of the sunny (Figure 2a) and the cloudy (Figure 2b) day. At midday on the sunny day, a point on a leaf at the top of the canopy experiences full sunlight (>1000 µmol m^−2^ sec^−1^) (Figure 2c), whereas all points lower in the canopy experience sun flecks (Figure 2e,g). Sunflecks are seen to be highly dynamic over the course of the day, particularly in the middle layers (Figure 2e). On an intermittently cloudy day, points on both the top leaves and those below are subject to large fluctuations in absorbed light (Figure 2d,f), whereas little light reaches points on the bottom layer (Figure 2f).

**Figure 3:**
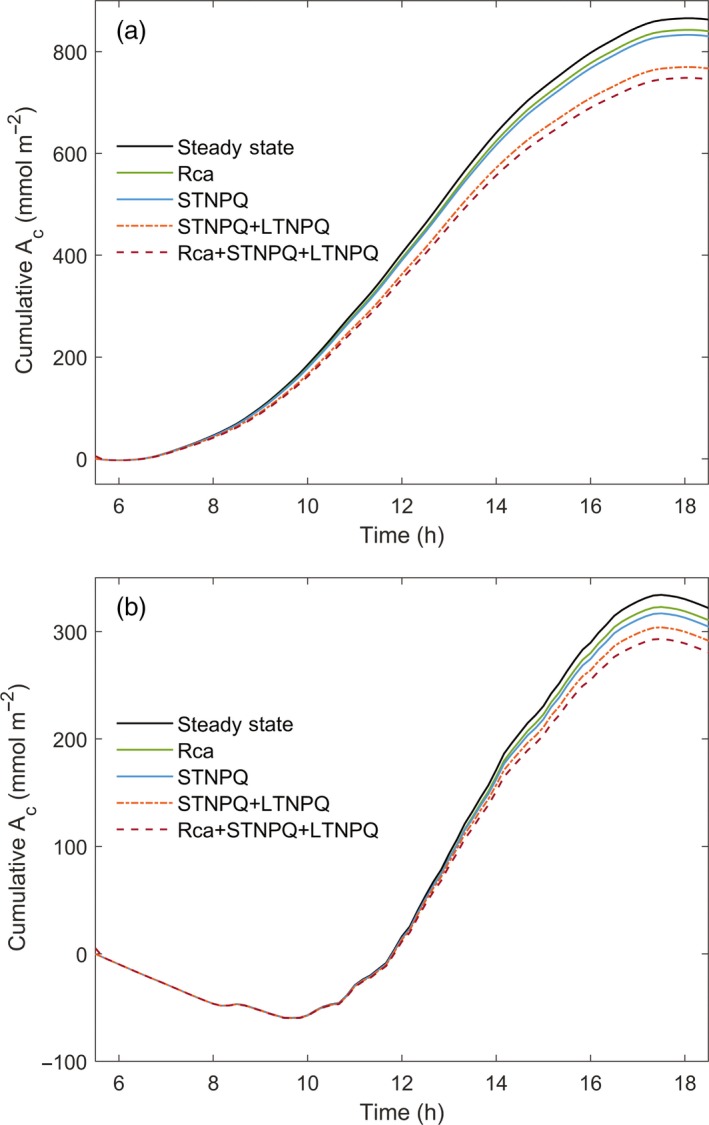
The cumulative net CO_2_ assimilation of the *Glycine max* (soybean) canopy of Figure 1 (*A_c_*) on the simulated sunny day (Figure 2a) and actual cloudy day (Figure 2b). The black line is the predicted assimilation of CO_2_ that would occur if photosynthesis responded instantaneously to changes in light, with no lags in efficiency. Rca accounts for the losses that result from the lags in the activation of Rubisco on shade–sun transitions. STNPQ accounts for the losses that result from lags in the both forms of NPQ. Rca + STNPQ + LTNPQ accounts for lags in both Rubisco activation and relaxation of NPQ. The simulations starts at dawn (05:30 h), with *A*_c_ = 0.

**Figure 4:**
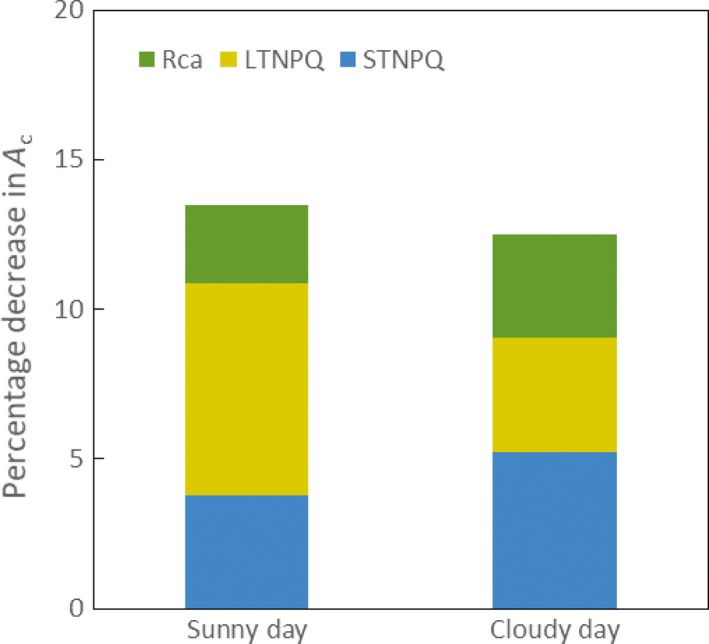
The percentage loss in potential daily canopy photosynthetic net carbon assimilation (*A_c_*) through losses of efficiency from lags in non‐photochemical quenching (NPQ) relaxation and Rubisco activation, calculated from the simulation presented in Figure 3. Blue bars (STNPQ) are the decreases in *A_c_* caused by short‐term NPQ relaxation; yellow bars (LTNPQ) are the decreases of *A_c_* caused by long‐term NPQ relaxation (Zhu *et al.*, 2004); green bars (Rca) are the predicted decreases of *A_c_* resulting from lags in Rubisco activation. The simulated sunny clear‐sky day is plotted on the left and the intermittently cloudy day is plotted on the right.

The net cumulative canopy assimilation (*A*_c_) was calculated over the daylight hours of the sunny (Figure 3a) and the cloudy (Figure 3b) day, driven by the simulated temporal and spatial dynamics of lighting for the whole canopy. Simulations of *A*_c_ compared instantaneous responses to light fluctuations with the slower responses resulting from the delays caused by photosynthetic induction (via Rubisco; Rca), STNPQ (including qE and qM) and LTNPQ. This quantified the loss of potential canopy carbon fixation caused by these delays. On the sunny day, a positive rate of net total carbon assimilation was observed at 07:00 h, and a positive rate of cumulative carbon gain continued until around 18:00 h, when canopy respiration was again predicted to exceed photosynthesis (Figure 3a). Simulated differences in cumulative assimilation resulting from delays in adjustment to light fluctuations become evident around 08:00 h, and progressively amplify in cumulative carbon gain through the daylight hours (Figure 3a). The total loss resulting from these delays is 13.5%, with LTNPQ accounting for the largest part of this loss (Figures 3a and 4). On the cloudy day, because the light intensity was very low in the morning (Figure 2b), the net assimilation was negative from dawn until 09:40 h, because total canopy respiration exceeded the photosynthesis until this time. Beyond this point, the simulated differences in cumulative net assimilation resulting from delays in adjustment to light fluctuations progressively amplified in cumulative net assimilation until 17:00 h (Figure 3b). The total loss as a result of these lags was 12.5%, with STNPQ accounting for the largest part of this loss (Figures 3 and 4). Although the simulated proportionate losses were similar on both days (Figure 4), the absolute loss on the intermittently cloudy day (40 mmol m^−2^) was less than half that on the sunny day (115 mmol m^−2^).

Partitioning the causes of simulated losses on the sunny day shows that resulting from LTNPQ was almost twice the loss resulting from STNPQ, and was approximately 2.7 times the loss caused by Rubisco activation (Figure 4). By contrast, on a cloudy day the loss resulting from STNPQ is higher, contributing about 40% of the total loss, whereas the loss resulting from Rubisco induction is similar to that resulting from LTNPQ (Figures 3b and 4).

**Figure 5:**
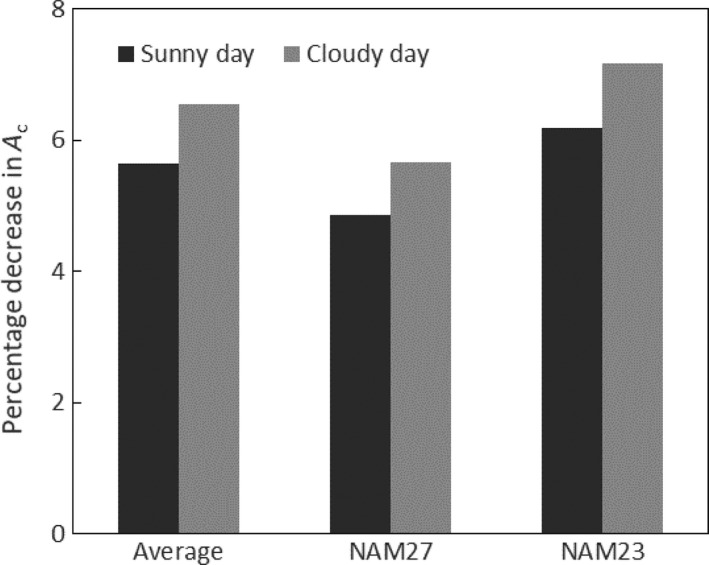
The effect of genetic variation in the speeds of short‐term non‐photochemical quenching (STNPQ) relaxation on losses of potential daily net CO_2_ assimilation (*A*_c_) in the *Glycine max* (soybean) canopy of Figure 1. Illustrated are losses in *A*_c_ resulting from the average of the measured rates of relaxation of STNPQ for the nested association mapping (NAM) soybean parent population and for the NAM lines showing the fastest (NAM27) and slowest (NAM23) rates of STNPQ relaxation. Dark‐gray bars are losses predicted for the sunny day (Figure 2a) and light‐gray bars are losses for the intermittantly cloudy day (Figure 2b).

To assess the potential impact of genotypic variation on assimilation, NPQ relaxation rates were measured for the 41 parental lines of the soybean NAM population. Variation in qE and qM across the NAM population were 40 and 49% between the slowest and fastest genotype, respectively. Values for these two genotypes and the average across all members of the NAM population were used in the model. The genotype with the fastest relaxation (NAM27) assimilated about 1.3% more CO_2_ across the day compared with the genotype with the slowest relaxation (NAM23), and assimilated approximately 0.8% more CO_2_ than average for the sunny day (Figure 5). On the cloudy day, the fastest genotype (NAM27) assimilated approximately 0.9% more than average (Figure 5), suggesting small but significant additive effects across a growing season.

**Figure 6:**
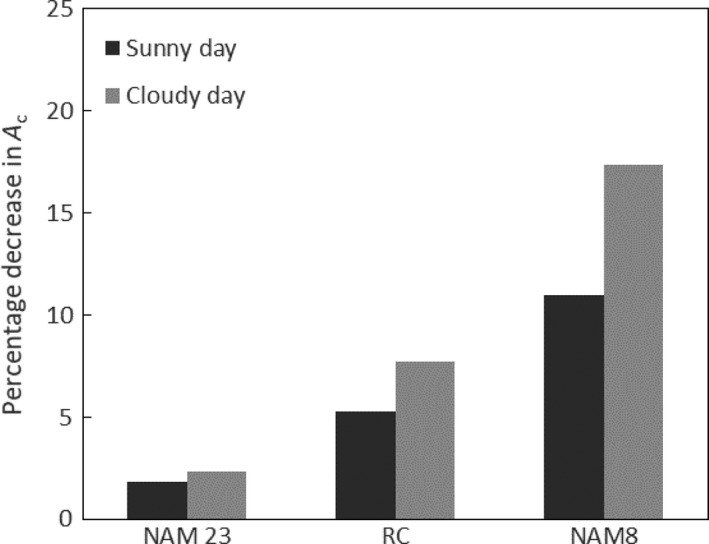
The effect of genetic variation in the speeds of Rubisco activation on losses of potential daily net CO_2_ assimilation (*A*_c_) in the canopy of Figure 1 using the daily sunlight profiles of Figure 2(a, b). Measurements from three *Glycine max* (soybean) genotypes were used (Soleh *et al.*, 2017): NAM23, which showed the fastest activation of Rubisco (τ_Rubisco_); RC, the average for the population; and NAM8, which showed the slowest activation. Dark‐gray bars are losses predicted for the sunny day (Figure 2a) and light‐gray bars are losses for the intermittantly cloudy day (Figure 2b).

Previously published measurements of photosynthetic induction in the NAM population showed large variation between genotypes in induction (Soleh *et al.*, 2017). The time course of Rubisco activation (*τ*_Rubisco_) for the two genotypes representing the slowest and fastest induction rates, and a third representing the middle of this range, were added here to the canopy model to simulate the variation in loss caused by the speed of the response on the sunny and the cloudy days (Figure 6). The loss in assimilation as a result of Rubisco activation is up to 17.4% for NAM8, which had the slowest Rubisco activation (*τ*_Rubisco_ = 1850.0 sec), whereas NAM23, the genotype with the fastest Rubisco activation (*τ*_Rubisco_ = 129.7 sec), reduced the loss to only 2.3% on the cloudy day and 1.9% on the sunny day.

## Discussion

Using a rendering of an actual fully developed field canopy (Figure 1), the present study suggests that the lower efficiency of photosynthesis during light fluctuations for an elite soybean cultivar costs about 13.5% of potential crop assimilation on both sunny and cloudy days in the critical pod‐filling phase (Figure 4). On the simulated clear‐sky day, LTNPQ appears to be the largest cause of this loss. On a cloudy day, photosynthetic induction and LTNPQ contribute equally, and the loss resulting from STNPQ contributes the most, at 40% (Figure 4). Crop biomass is about 40% carbon. Assuming then that one mole of CO_2_ assimilated, net of respiration, results in 75 g of biomass, then the growth loss resulting from lags in the adjustment of photosynthesis to light fluctuations in the canopy would be a substantial 90 kg ha^−1^ day^−1^ on a sunny day. With no mechanisms known that could allow for the instantaneous induction of photosynthesis on shade–sun transitions or the relaxation of NPQ on sun–shade transitions, it appears unlikely that breeding or bioengineering could recover more than about half of this loss. Nevertheless, a 6.5% increase in net photosynthetic efficiency, if translated into increased crop yields, would be exceptional. It could either provide a key part of the anticipated future need for increased yield potential to ensure global food security or, should food demand stabilize, serve to reduce the global footprint of arable agriculture (Long *et al.*, 2015). How could these losses in fluctuating light be decreased?

The engineered acceleration of STNPQ relaxation has already been undertaken in tobacco by upregulating the levels of the two enzymes involved in the interconversion of violaxanthin and zeaxanthin and the photosystem‐II protein PsbS, which affects the amplitude of NPQ. This increased the quantum yield of CO_2_ assimilation in fluctuating light and the rate of whole‐chain electron transport by approximately 50%. In replicated field trials, biomass production was increased by 14–21%; however, this was achieved with a 50‐ to 100‐fold increase in the two enzymes and an approximately fivefold increase in PsbS (Kromdijk *et al.*, 2016). It would seem unlikely that this scale of diversity exists within the germplasm of a crop, suggesting that improvements on this scale might only be achieved by the transgenic addition of extra copies of the relevant genes or by the engineering of promoter regions to upregulate expression. This might be confirmed by the results obtained here with the NAM population parent lines, where the gain achieved with the fastest STNPQ‐relaxing line showed only a 1% improvement over the average in terms of *A*_c_ (Figure 5). Although small, this could still have value. In 2017 soybean occupied 124 Mha of the global surface, which produced 353 Mt of beans (FAOStat, 2017). As such, a 0.8% increase could mean an additional 2.8 Mt or it could release over 1 Mha of land no longer needed for soybean production. Importantly, this increase, although small in relative terms, can potentially be gained by conventional breeding.

Given that the relaxation of LTNPQ is the largest contributor to losses on a sunny day, this suggests that the largest gains may be achieved by focusing on this aspect. Several genes, in particular those involved in the repair and replacement of the photosystem‐II D1 protein, have been functionally implicated in the slow phase of NPQ recovery (Nixon *et al.*, 2010). Although the optimal targets in crops remain unclear, it will be important to see these tested in model plants. With the rapid growth of complete genomic sequences for many accessions of major crops, genome‐wide association analysis provides an approach to reveal further targets (Wang *et al.*, 2017a).

What factors limit the speed of induction on a shade–sun transition? In the chloroplast, photosynthesis is limited by the build‐up of Calvin cycle intermediates, in particular the CO_2_ acceptor molecule ribulose 1,5‐bisphoshate, and the induction of light‐activated photosynthetic enzymes, in particular the activation of Rubisco by Rca. Sufficient accumulation of ribulose 1,5‐bisphoshate is assumed to require approximately 60 sec, whereas the activation of Rubisco may require more than 10 min (Mott and Woodrow, 2000; Taylor and Long, 2017). At the leaf level, induction can be limited by slow stomatal opening, where full opening can require many minutes (McAusland *et al.*, 2016; De Souza *et al.*, 2019; Faralli *et al.*, 2019; Acevedo‐Siaca *et al.*, 2020), and by mesophyll conductance, which generally increases with incident light. The rate of increase in mesophyll conductance upon induction is generally considered faster than both stomatal opening and Rubisco activation (Deans *et al.*, 2019), but the variability between species and environmental conditions is not well defined. Several studies have inferred from both modeling and the *in vivo* estimation of Rubisco activity (*V*_c,max_) that the activation of Rubisco is the key limitation to the speed of induction (Mott and Woodrow, 2000; Yamori *et al.*, 2012; Soleh *et al.*, 2016; Soleh *et al.*, 2017; Taylor and Long, 2017). This gains support from the observation that in wheat and soybean, intercellular [CO_2_] is higher during induction than at steady state (Soleh *et al.*, 2017; Taylor and Long, 2017), whereas the converse would be expected if stomatal opening was the dominant factor. In other species the speed of stomatal opening appears to be the dominant limitation limiting the speed of induction (McAusland *et al.*, 2016; De Souza *et al.*, 2019), and this may be accentuated under stress conditions (Faralli *et al.*, 2019). In the current study, only the activation of Rubisco by Rca was considered, so the losses resulting from slow induction must be considered a minimum, where slow increases in stomatal and mesophyll conductance could exacerbate these losses. There is already evidence that increasing the activity of Rca by the upregulation of expression increases the speed of activation (Yamori *et al.*, 2012), whereas site‐directed mutagenesis has been shown as a non‐transgenic means to increase Rca activity (Perdomo *et al.*, 2019). In addition, significant natural variation in the speed of induction has been demonstrated within the germplasm of cassava, soybean and wheat (Soleh *et al.*, 2017; De Souza *et al.*, 2019; Salter *et al.*, 2019), providing further non‐transgenic opportunities for decreasing the losses resulting from reduced photosynthetic efficiency during induction. Simulation of this variation in our canopy model suggests that considerable gains could be achieved in soybean by conventional breeding that selects for faster rates of induction (Figure 6).

The purpose of this work was to highlight the potential for improving crop photosynthesis under non‐steady‐state lighting conditions. To do this we had to draw on disparate sources for model parameterization, as not all parameters were available for soybean. Additionally, some of the parameters used were measured using different protocols, such as NPQ relaxation kinetics of soybean cv. LD11‐2170 and the NAM population. Therefore, the predictions here should be taken only as an indication of opportunity, and need to be further improved by better measurements and parametrization. First, the rate constant of the NPQ relaxation is possibly related to light intensities or light‐intensity differences (Dall'Osto *et al.*, 2014). Second, it is not clear how leaf position and age influence the NPQ relaxation speed. Third, the long‐term NPQ responses of soybean and most major crops have not been well defined, and the correlation between LTNPQ and the accumulated light input has not been validated with sufficient data. Fourth, the correlation between Rubisco activation kinetics and light intensity is poorly defined. Thus more integrated measurement and analysis of NPQ relaxation, Rubisco activation kinetics, stomatal opening and mesophyll conductance dynamics in the major crops will improve the model prediction and ability to partition causes. Finally, the lighting was simulated for 5‐mm^2^ pixels of the leaf surface; however, NPQ activation and Rubisco activation occur at the level of the chloroplast, with a cross‐sectional area of about 25 µm^2^. As a result, the speed of the light change at an individual chloroplast with the change in solar angle will be faster than that simulated. Simulating smaller areas greatly increases the computational time, but is likely to become practical as computational power increases. The compromise used here will have resulted in some underestimation of the cost of these light transitions, however.

In conclusion, this first model analysis of light fluctuations in a rendering of an actual crop canopy indicates unexploited breeding and bioengineering targets to substantially improve photosynthetic efficiency and productivity. Although this analysis is limited to soybean, it appears reasonable to expect similar gains in other C3 crops, including cassava, wheat and rice. Although we cannot be certain that an improvement in photosynthetic efficiency will increase the yield, there is evidence for all four crops that an artificial increase in leaf photosynthesis by season‐long elevation of [CO_2_] under open‐air field conditions results in highly significant increases in yield (Long *et al.*, 2006; Rosenthal *et al.*, 2012; Hasegawa *et al.*, 2013).

## Experimental Procedures

### 3D soybean model and light distribution simulations

The dynamics of lighting within a soybean canopy were predicted with a 3D architectural representation, using our previously presented framework for crop canopies (Song *et al.*, 2013; Wang *et al.*, 2017b). The model was parameterized on the measured architecture of a soybean crop (*G. max* L. Merr., Pioneer 93B15) on the University of Illinois South Farms. The leaf lengths, widths, petiole lengths and angles for trifoliate leaves of soybean (Aug 20) were obtained from Song *et al*., (2019). The canopy had a row spacing of 38 cm and a plant spacing within the rows of 10 cm. The model divides the surface of each leaf into ‘pixels’ of approximately 5 mm^2^ in area, which is much smaller than the ‘pixels’ used in Song *et al.*, (2019; about 1 cm^2^). To estimate light changes in the canopy, a forward ray‐tracing algorithm (fasttracer; Song *et al.*, 2013; Wang *et al.*, 2017b; Townsend *et al.*, 2018; Song *et al.*, 2019) was used to predict the light absorption of each 5‐mm^2^ pixel every 10 sec from 05:00 to 19:00 h on 20 August 2019 in Champaign, IL, USA (40.11 N, 88.21 W). At each time point the direct and diffuse light entering and within the canopy is predicted, together with the scattered radiation through reflection from, and transmission through, the leaves within the canopy. Leaf reflectance and transmission were both set to 0.075 (Song *et al.*, 2013). The sum of the direct, diffuse and scattered light incident at each pixel, less the reflectance and transmission, gave the absorbed photosynthetically active photon flux. Atmospheric transmittance was set as 0.85 to estimate the incident direct and diffuse light reaching the top of the canopy at each time point on a sunny day (Song *et al.*, 2013). A partially cloudy day was simulated by using the measured direct and diffuse light on 20 August 2018 in Bondville, IL, USA (SURFRAD, 2019). For each time point (*t*), the light absorption of each 5‐mm^2^ leaf pixel on the cloudy day, *I*_l_c_(*t*), was calculated as:

(1) $$I_{\text{l\textbackslash{}\_c}}\left(t\right)=I_{\text{l\textbackslash{}\_s}}\left(t\right)\frac{Q_{\text{in\textbackslash{}\_c}}\left(t\right)}{Q_{\text{in\textbackslash{}\_s}}\left(t\right)},$$

where *I*_l_s_(*t*) is the light absorption of each leaf pixel on the sunny day, which was calculated by the ray‐tracing algorithm. *Q*_in_s_(*t*) is the incident light above the canopy on the sunny day and *Q*_in_c_(*t*) is the incident light above the canopy on the cloudy day, based on the actual incident light measured.

### Simulation of dynamic photosynthesis

Dynamic photosynthetic rates were calculated for every 10 sec (Δ*t*) of the day using the absorbed light for each 5‐mm^2^ pixel, considering the rates of Rubisco activation and NPQ relaxation. Rubisco deactivation was also taken into account, as the extent of the deactivation during a period of shade affects the duration of the induction following a return to higher light levels. The *in vivo* dynamics of Rubisco activation were calculated using the Rca model of Mott and Woodrow (2000). The steady‐state maximum Rubisco activity (*V_c_*_max0__ *_s_*) was related to the level of Rca (70 mg m^−2^) (Mott and Woodrow, 2000), where *K_a_*_Rca_ is a constant in determining the maximum steady‐state activity of Rubisco (Table 1):

(2) $$V_{c\max0\_s}=\frac{V_{c\max0}\left[\text{Rca}\right]}{k_{a_{\text{Rca}}}+\left[\text{Rca}\right]}$$

**Table 1:** Input parameters of the *Glycine max* (soybean) canopy model

Parameter	Full name	Value	Reference
*C_i_*	Intercellular CO_2_ concentration	280 μbar	Environmental input
*f_h_*	Empirical constant in determining the decrease of *F*_v_/*F*_m_ for a given *I*_int_.	5.1e+5	Zhu *et al.* (2004)
*F*v*/F*m	Maximum quantum yield of photosystem II	0.801	Measured
Γ*	The CO_2_ compensation point in the absence of dark respiration	38.6 μbar	Von Caemmerer (2000)
*J*_max_	Maximum electron transport capacity	200 μmol m^−2^ sec^−1^	Bernacchi *et al.* (2005)
*K*_o_	Michaelis–Menten constant of Rubisco for O_2_	404 mbar	Von Caemmerer (2000)
*K*_c_	Michaelis–Menten constant of Rubisco for CO_2_	248 μbar	Von Caemmerer (2000)
*k_a_*_Rac_	A constant for the calculation of maximum steady‐state Rubisco activity (eqn 2)	12.4 mg m^−2^	Mott and Woodrow (2000)
*k*_light_	A constant for the calculation of steady‐state Rubisco activity (eqn 3)	120 μmol m^−2^ sec^−1^	Sage and Seemann (1993)
*K*_TaoRac_	A constant for the calculation of the time constant of Rubisco activation	214 min mg m^−2^	Mott and Woodrow (2000), Soleh *et al.* (2016)
O_2_	Intercellular O_2_ concentration	210 mbar	Environmental input
*PqE*	Proportion of the contribution of *qE* in steady‐state ΦNPQ	0.7	Measured
*PqM*	Proportion of the contribution of *qM* in steady‐state ΦNPQ	0.3	Measured
[Rac]	Concentration of Rubisco activase	70 mg m^−2^	Mott and Woodrow (2000)
*R*_d0_	Dark respiration	1.2 μmol m^−2^ sec^−1^	Measured
Temp	Leaf temperature	25°C	Environmental input
τ*_qE_*	Time constant of *qE* relaxation	0.56 min	Measured
τ*_qM_*	Time constant of *qM* relaxation	16.8 min	Measured
*V*_max0_	Maximum Rubisco activity	120 μmol m^−2^ sec^−1^	Bernacchi *et al.* (2005)

The maximum Rubisco activity (*V_c_*_max_) was calculated by the following equations:

(3) $$V_{\max\_s}\left(t\right)=V_{c\max0\_s}\frac{I_{l}\left(t\right)}{k_{\text{light}}+I_{l}\left(t\right)},$$

(4) $$A_{v\max}\left(t\right)=A_{v\max}\left(t-\Delta t\right)-\Delta t\cdot \frac{\left(A_{v\max}\left(t-\Delta t\right)-V_{\max\_s}\left(t\right)\right)}{\tau_{\text{Rubisco}}},$$

(5) $$V_{c\max}\left(t\right)=A_{v\max}\left(t\right)\cdot V_{c\max\_0},$$

where *t* is time (s), *I_l _*(*t*) is the absorbed light of each leaf unit at time *t* and *K*_light_ are constants (Table 1). τ_Rubisco_ is the time constant of Rubisco activation and deactivation; here, τ_Rubisco_ is 180 sec for Rubisco activation and 300 sec for Rubisco deactivation (Taylor and Long, 2017). *A*_*v*max_ *(t)* is the activated proportion of Rubisco.

The short‐term relaxation kinetics of non‐photochemical quenching (STNPQ) was defined as the dynamics of NPQ relaxation over the first 30 min, and is described by a bi‐exponential curve (Dall'Osto *et al.*, 2014). Here, qE is the fast relaxing energy‐dependent quenching, whereas qM combines all of the remaining processes up to 30 min:

(6) $$\Phi_{\mathrm{qE}}\left(t\right)=\Phi_{\mathrm{qE}}\left(t-\Delta t\right)-\Delta t\cdot \frac{\left(\Phi_{\mathrm{qE}}\left(t-\Delta t\right)-\Phi_{\text{NPQs}}\left(t\right)\cdot P_{\mathrm{qE}}\right)}{\tau_{\mathrm{qE}}}$$

(7) $$\Phi_{\mathrm{qE}}\left(t\right)=\Phi_{\mathrm{qE}}\left(t-\Delta t\right)-\Delta t\cdot \frac{\Phi_{\mathrm{qE}}\left(t-\Delta t\right)-\Phi_{\text{NPQs}}\left(t\right)\cdot P_{\mathrm{qE}})}{\tau_{\mathrm{qE}}}$$

(8) $$\Phi_{\mathrm{qM}}\left(t\right)=\Phi_{\mathrm{qM}}\left(t-\Delta t\right)-\Delta t\cdot \frac{\left(\Phi_{\mathrm{qM}}\left(t-\Delta t\right)-\Phi_{\text{NPQs}}\left(t\right)\cdot P_{\mathrm{qM}}\right)}{\tau_{\mathrm{qM}}}$$

(9) $$\Phi_{\text{NPQ}}\left(t\right)=\Phi_{\mathrm{qE}}\left(t\right)+\Phi_{\mathrm{qM}}\left(t\right)$$

where Φ_NPQs_ is the steady state *Φ*_NPQ_ obtained from the measured light‐response curves (see next section) of soybean (cv. LD11‐2170). *N*_1_ and *N*_0_, which are set to 0.00028 and 0.0371, are constants calculated from the measured *Φ*_NPQs_. *P_qE_* and *P_qM_* are the proportions of *qE* and *qM*. *τ* *_qE_* and *τ* *_qM_* are the time constants of *qE* and *qM* relaxation, respectively.

Long‐term non‐photochemical quenching (LTNPQ), which includes, among other terms, photoinhibitory quenching (qI) and photoinhibition‐independent sustained quenching (qH) decreases the maximum quantum yield of photosystem II (PSII) and in turn the maximum quantum yield of CO_2_ uptake (Zhu *et al.*, 2004). Following Zhu *et al. *(2004), the LTNPQ was correlated with a weighted light dose, and the change in the maximum quantum yield of PSII photochemistry, *A_Fv/Fm_*(*t*), was calculated as:

(10) $$T_{f}=0.0033T^{2}-0.1795T+3.4257,$$

(11) $$I_{\text{int}}\left(t\right)=\sum_{i=1}^{i=60}\frac{I_{l}\left(t-i\right)}{\left(1-\frac{i-1}{60}\right)},$$

(12) $$A_{Fv/Fm}\left(t\right)=1-I_{\text{int}}\left(t\right)\cdot \frac{T_{f}}{f_{h}},$$

where *I*_int_ is the weighted light dose at time t. *T_f_* is an empirical factor relating the relative decrease of *Fv/Fm* to temperature (*T*), and *T* was set as 25°C. *f_h_* is an empirical constant used in determining the decrease of *Fv/Fm* for a given *I*_int_. For cold‐susceptible species, *f_h_* is 5.13.

The quantum yield of PSII was calculated as:

(13) $$\Phi_{\text{PSII}}=A_{\mathrm{Fv}/\mathrm{Fm}}\left(t\right)\cdot \mathrm{Fv}/\mathrm{Fm}-\Phi_{\text{NPQ}}\left(t\right).$$

The electron transport rate, and in turn the rate of leaf CO_2_ uptake (*A*), is limited by the quantum yield of PSII, incident light and maximum electron transport capacity (*J*_max_):

(14) $$J=\min\;\left(\frac{1}{2}\Phi_{\text{PSII}}I\left(t\right),J_{\max}\right).$$

The rate of leaf CO_2_ uptake (*A*) at time *t* was simulated by the following equations:

(15) $$A_{j}\left(t\right)=\frac{\left(C_{i}-\Gamma^{*}\right)J\left(t\right)}{\left(4C_{i}+9.3\Gamma^{*}\right)}-R_{d},$$

(16) $$A_{c}\left(t\right)=\frac{V_{c\max}\left(t\right)\left(C_{i}-\Gamma^{*}\right)}{\left(C_{i}+K_{c}\left(\right.1+\frac{O_{2}}{K_{o}}\right)}-R_{d},$$

(17) $$A\left(t\right)=\min(A_{j}\left(t\right),A_{c}\left(t\right)),$$

where *R*_d_ is the leaf respiration. As lower canopy leaves respire less than upper canopy leaves, *R*_d_ was scaled with the overlying leaf area index (LAI), as described previously (Srinivasan *et al.*, 2017):

(18) $$R_{d}\left(z\right)=R_{d0}\exp\left(-\mathrm{kn}\cdot \text{LAI}\left(z\right)\right),$$

where *R*_d0_ is the respiration of the uppermost leaf layer with a measured value of 1.2 (±0.22) for soybean (cv. LD11‐2170) and *kn* is an exponential extinction coefficient with a measured value of 0.2 (Srinivasan *et al.*, 2017).

Then the canopy net CO_2_ uptake (*A*_c_) was calculated as:

(19) $$A_{c}\left(t\right)=\frac{\sum \left(A_{i}\left(t\right)\cdot S_{i}\right)}{S_{\text{ground}}},$$

where *A_i_*(*t*) is the CO_2_ uptake rate of a leaf pixel, *S_i_* is the surface area of each pixel and *S*_ground_ represents the occupied ground area of the simulated canopy. All simulations were conducted in matlab 2017 (Mathworks®, https://uk.mathworks.com).

### Measurement of dynamic NPQ parameters

To determine the kinetics of STNPQ relaxation in soybean (parameters listed in Table 2), chlorophyll fluorescence and gas exchange were measured during the transition from high light (1800 μmol m^−2^ sec^−1^) to low light (200 μmol m^−2^ sec^−1^). Measurements were taken on 9 March 2019 in a controlled‐environment glasshouse at the University of Illinois at Urbana‐Champaign. The air temperature inside the glasshouse was set as 28°C (day)/24°C (night). Leaf CO_2_ uptake and modulated chlorophyll fluorescence of the youngest fully expanded leaf was measured on 30‐day‐old soybean plants (cv. LD11‐2170) with a gas‐exchange system incorporating a controlled‐environment leaf cuvette with a head containing a modulated chlorophyll fluorometer and an LED light source (LI‐6400XT and LI‐6400‐40; LI‐COR, https://www.licor.com). The measurements were made on six replicate plants. The leaves were first acclimated to dark for 30 min, with a leaf cuvette temperature (*T*_block_) of 28°C and a [CO_2_] of 400 µmol mol^−1^; the light intensity was then increased to 1800 μmol m^−2^ sec^−1^ for 30 min and then decreased to 200 μmol m^−2^ sec^−1^ for the next 30 min, to simulate a sun–shade transition. Leaf CO_2_ exchange and modulated chlorophyll fluorescence were recorded before the light was turned on, and then every 60 sec for the following 30 min. The measured time series data of ΦNPQ changes, represented by the following equation (Dall'Osto *et al.*, 2014):

(20) $$\Phi_{\text{NPQ}}=\Phi_{\mathrm{qI}}+\Phi_{\mathrm{qE}}e^{\left(-t/\tau qE\right)}+\Phi_{\mathrm{qM}}e^{\left(-t/\tau qM\right)},$$

were fitted with a polynomial (fit function; matlab 2017). The fitted values are listed in Table 2.

**Table 2:** Measured non‐photochemical quenching (NPQ) relaxation parameters of *Glycine max* (soybean) cv. LD11‐2170

Parameters	Soybean
*F*_v_ */F*_m_	0.801 ± 0.032
Φ*_qE_*	0.318 ± 0.016
Φ*_qI_*	0.097 ± 0.015
Φ*_qM_*	0.066 ± 0.008
τ*_qE_* (min)	0.558 ± 0.051
τ*_qM_* (min)	16.826 ± 9.134

For light‐response curves, the leaf was dark‐adapted for 30 min and then acclimated to a light intensity of 1500 µmol m^−2^ sec^−1^ and a CO_2_ concentration of 400 µmol mol^−1^ inside the cuvette. After 20 min the chamber light was varied according to the following sequence: 2000, 1500, 1000, 500, 300, 200, 100 and 50 µmol m^−2^ sec^−1^. Chlorophyll fluorescence and gas‐exchange measurements were recorded after the conditions inside the cuvette regained stability at each light level, the minimal time interval was 5 min. Chlorophyll fluorescence measurements were used to calculate steady‐state NPQ (*Φ*_NPQs_, eqn 6).

### Variation in the rate of recovery of STNPQ quenching across the parents of the soybean NAM population

The 41 parents of the soybean NAM population (Song *et al.*, 2017; Diers *et al.*, 2018) were planted on the South Farms of the University of Illinois at Urbana‐Champaign, on 6 June 2019. The parents were planted in 1.2‐m‐long single‐row plots with a 0.75‐m row spacing. The experiment was arranged with a randomized complete block design with five replicate plots per genotype. On the 26 July 2019, when plants were at the R1 developmental stage, three 0.48‐mm leaf disks were collected from the uppermost mature leaf of each replicate plot in the field and floated on water in 24‐well plates for transport back to the laboratory. Leaf disks were then laid adaxial side up on square Petri dishes containing damp filter paper, sealed, wrapped in aluminum foil and incubated overnight at 25ºC to allow for the complete relaxation of NPQ. The next day, the modulated PSII chlorophyll fluorescence of the disks on each Petri dish was imaged (FluorCam FC 800‐C; PSI‐Photon Systems Instruments, Drasov, Czech Republic, https://psi.cz/). Disks were exposed to 10 min of approximately 1000 μmol m^−2^ sec^−1^ white light (6500 K) to induce NPQ and then 50 min of darkness. Saturating pulses (4000 μmol m^−2^ sec^−1^ white light) to determine *F*_m_ were given at 9, 40, 60, 80, 100, 120, 160, 200, 240, 300, 360, 420, 480, 540 and 598 sec after the actinic light was turned on, and at 1, 2, 4, 6, 8, 10, 14, 18, 22, 26, 32, 38, 44 and 50 min after the actinic light was turned off. The background was excluded automatically and NPQ values at each pulse were calculated. The maximum NPQ value for each leaf disk was set to 1, with other values normalized relative to this. Relaxation of NPQ with time was fitted by nonlinear least squares to equation 19 (nls function, R‐project).

### Simulating the effect of variation in photosynthetic induction across the NAM parent lines

Measured time courses of Rubisco (*V_c_*_max_) activation during photosynthetic induction were extracted from Soleh *et al. *(2017) for the NAM parent lines, using image‐capture and analysis software (getdata graph digitizer, obtained from S. Federov, http://getdata-graph-digitizer.com). Fifteen points were captured per curve for each leaf disk and then fitted to an exponential function:

(20) $$V_{c\max}=ae^{\left(-t/\tau_{\text{Rubisco}}\right)}+c,$$

where τ_Rubisco_ is the time constant of Rubisco activation, *c* is the Rubisco activity in the dark and *a* is the increase of Rubisco activity from dark to light. The fitted values are listed in Table 3. Then the measured τ_Rubisco_ values for NAM23, RC and NAM8 were used in this simulation. The time constant of Rubisco deactivation was assumed to be double the time required for the activation of each genotype (Taylor and Long, 2017).

**Table 3:** Rubisco activation parameters estimated by using the time course of maximum carboxylation capacity of three selected parents of the *Glycine max* (soybean) nested association mapping (NAM) population during the induction response (Soleh *et al.*, 2017)

Parameters	NAM23	RC	NAM8
*a* (µmol m^−2^ sec^−1^)	72.2	80.0	85.0
*c* (µmol m^−2^ sec^−1^)	21.6	17.3	7.6
τ_Rubisco_ (sec)	129.7	471.7	1850.0
*R* ^2^	0.982	0.977	0.976

## Author Contributions

SPL, YW and SJB designed the study. YW performed the computational analysis. YW, EMDB and SJB conducted the STNPQ measurements. SPL, YW, SJB and EMDB wrote the article.

## Conflict of Interest

The authors declare that they have no conflicts of interest.

## Data Availability

The code and data are available at https://doi.org/10.13012/B2IDB-9453481_V1.
